# Downregulation of Cell Cycle and Checkpoint Genes by Class I HDAC Inhibitors Limits Synergism with G2/M Checkpoint Inhibitor MK-1775 in Bladder Cancer Cells

**DOI:** 10.3390/genes12020260

**Published:** 2021-02-11

**Authors:** Michèle J. Hoffmann, Sarah Meneceur, Katrin Hommel, Wolfgang A. Schulz, Günter Niegisch

**Affiliations:** Department of Urology, Medical Faculty, Heinrich-Heine-University Duesseldorf, Moorenstr. 5, 40225 Duesseldorf, Germany; meneceur@hhu.de (S.M.); kahom101@hhu.de (K.H.); wolfgang.schulz@hhu.de (W.A.S.); guenter.niegisch@med.uni-duesseldorf.de (G.N.)

**Keywords:** bladder cancer, cell cycle gene, cell cycle arrest, HDAC, epigenetic inhibitor, Romidepsin, MK- 1775, G2/M checkpoint, mutation, copy number variation

## Abstract

Since genes encoding epigenetic regulators are often mutated or deregulated in urothelial carcinoma (UC), they represent promising therapeutic targets. Specifically, inhibition of Class-I histone deacetylase (HDAC) isoenzymes induces cell death in UC cell lines (UCC) and, in contrast to other cancer types, cell cycle arrest in G2/M. Here, we investigated whether mutations in cell cycle genes contribute to G2/M rather than G1 arrest, identified the precise point of arrest and clarified the function of individual HDAC Class-I isoenzymes. Database analyses of UC tissues and cell lines revealed mutations in G1/S, but not G2/M checkpoint regulators. Using class I-specific HDAC inhibitors (HDACi) with different isoenzyme specificity (Romidepsin, Entinostat, RGFP966), cell cycle arrest was shown to occur at the G2/M transition and to depend on inhibition of HDAC1/2 rather than HDAC3. Since HDAC1/2 inhibition caused cell-type-specific downregulation of genes encoding G2/M regulators, the WEE1 inhibitor MK-1775 could not overcome G2/M checkpoint arrest and therefore did not synergize with Romidepsin inhibiting HDAC1/2. Instead, since DNA damage was induced by inhibition of HDAC1/2, but not of HDAC3, combinations between inhibitors of HDAC1/2 and of DNA repair should be attempted.

## 1. Introduction

Urothelial carcinoma (UC) of the bladder constitutes a common cancer type and comprises 90% of cases with bladder cancer in industrialized countries [1]. Two-thirds of UC patients diagnosed with non-muscle invasive tumors (NMIBC) experience a rather mild clinical course with a low risk of disease progression needing regularly tumor resections. In contrast, patients with muscle-invasive tumors (MIBC) face a poor prognosis due to often rapid local and systemic progression [2]. Radical cystectomy accompanied by (neo-) adjuvant chemotherapy or recently approved modern immunotherapy with immune checkpoint inhibitors are standard of care. Chemotherapy of MIBC is based on combinations of cisplatin with other cytotoxic drugs since more than 30 years. Even though about 30–50% of patients respond initially, the majority of patients will progress or become refractory to treatment leading to a five-year survival below 30–40% [3,4]. Even with the advent of novel immunotherapeutic treatments, outcomes have improved only marginally [5,6]. Thus, new therapeutic approaches are urgently needed to improve patient survival.

Chromatin regulators and histone-modifying enzymes are exceptionally frequently mutated in UC, suggesting that epigenetic changes drive urothelial tumorigenesis and cancer progression [7,8]. Other epigenetic enzymes like histone deacetylases (HDAC) are frequently upregulated [9,10]. Since epigenetic modifications can be influenced by pharmaceutical inhibitors, they constitute new rational therapeutic targets for treatment of UC [11,12]. So-called pan-HDAC inhibitors (HDACi), like Vorinostat (SAHA)—targeting various isoenzymes of the four different HDAC classes—exhibit therapeutic efficacy and were approved for the treatment of hematological malignancies [13]. HDAC inhibition is generally thought to re-induce expression of genes repressed in cancers, prominently tumor suppressor genes that affect cell growth and survival of cancer cells [14]. However, we previously observed that Vorinostat has a rather low anti-neoplastic activity against UC cells [15]. Thus, we profiled expression and function of various HDAC isoenzymes in UC and identified class I HDACs, with the exception of HDAC8, as the most promising targets for HDACi [15,16,17,18].

Class I HDACs comprising the isoenzymes HDAC1, 2, 3, and 8 are known to promote cellular proliferation in tumors, while preventing differentiation and apoptosis [14]. In previous work we observed divergent effects of treatment with HDACi on the cell cycle of UC cells. In contrast to other cancer cell types that responded with G1 arrest [19,20,21,22,23,24,25], we observed significant accumulation of UC cells in the G2/M-phase following treatment with the class I-specific HDACi Romidepsin. Furthermore, treated UC cells displayed mitotic disturbances and underwent cell death, albeit to a limited extent and not exclusively by caspase-dependent apoptosis. Normal control cell lines like HBLAK and HEK-293 were much less affected by class I-HDACi like Romidepsin. We reported earlier that long-term proliferation was not significantly reduced by Romidepsin in these control lines. Concurringly, cell cycle changes were limited and cells did not accumulate in the G2/M phase. Furthermore, caspase activity remained unaltered [15]. 

Preliminary data suggested that inhibition of HDAC1 and HDAC2 together may exert different effects from inhibition of HDAC3 [15,18]. HDAC1/2 and HDAC3 are components of different transcription-corepressor complexes. While HDAC1 and HDAC2 can be components of CoREST, Sin3 or NuRD complexes, HDAC3 rather acts as a part of N-CoR and SMRT complexes [26,27]. Concurringly, HDAC1 and HDAC2 may have other distinct functions from HDAC3, e.g., in DNA damage signaling [28]. In previous work, we also noted a compensatory feedback loop between HDAC1 and HDAC2, but not for HDAC3 [15]. 

Thus, in this study we characterized the effects of three class I-specific HDACi with different isoenzyme specificities on genes regulating the cell cycle in more detail to elucidate different functions of the isoenzymes HDAC1/2 and HDAC3 in UC cells and to identify suitable compounds for combination treatments enhancing the anti-neoplastic effect of class I-HDACi. While Romidepsin, also named Depsipeptide or FK228, is a potent inhibitor of HDAC1 and HDAC2, Entinostat (MS-275) mainly inhibits HDAC1 and HDAC3. Both compounds have been studied in vitro and also in clinical studies [29,30,31]. We further used RGFP966 to specifically target HDAC3 only [32].

Initially, to understand the differences in the effect of HDACi on cell cycle arrest in UC cells and other cancer types, we performed in silico analyses of public data on genetic alterations in UC cell lines and patient tissues to clarify which cell cycle and checkpoint control genes are frequently mutated in UC and might thus explain the different response of our cells. We found genetic changes to be limited to relatively few genes of interest in our context, mainly well-known driver genes like *TP53*, *CDKN2A* (encoding for p16^INK4A^), *RB1* etc. Importantly, G2/M checkpoint genes were not mutated in the investigated cell lines so that this checkpoint should be functional. Accordingly, we speculated that targeting this checkpoint by the WEE1-inhibitor MK-1775 might enhance the efficacy of HDACi. 

We also speculated that the different response of UC cells to HDACi might not originate from cancer-specific genetic changes, but rather be caused by UC-specific functions of individual HDAC isoenzymes in the regulation of cell cycle and checkpoint control genes. Indeed, we found that inhibition of HDAC1 and HDAC2—but not of HDAC3—resulted in G2 arrest and that cell death might be induced via DNA damage that may not be recognized and repaired due to downregulation of DNA damage signaling components. G2/M checkpoint protein expression was altered by HDACi in a UC-specific manner so that the G2/M checkpoint inhibitor MK-1775 did not act synergistically in combination with the HDACi Romidepsin. 

## 2. Materials and Methods 

### 2.1. Cell Culture

Urothelial carcinoma cell lines (UCC) VM-CUB1 and UM-UC-3 were obtained from the DSMZ (Braunschweig, Germany) and Dr. B. Grossman (Houston, TX, USA). Cell lines were regularly verified by DNA fingerprint analysis and checked for mycoplasm contamination. Control cells comprised the spontaneously immortalized normal human urothelial cell line HBLAK [33] kindly provided by CELLnTEC, Bern, Switzerland. Cells were cultured as described previously [34]. 

HDAC inhibitors were purchased from Selleckchem (Houston, TX, USA), dissolved in DMSO and added to the cells 24 h after cell seeding for up to 72 h. Solvent control cells were treated with corresponding DMSO concentrations. Cell viability was measured by MTT assay (Sigma-Aldrich, St. Louis, MO, USA) as described previously [35]. 

### 2.2. Flow Cytometry

Cell cycle and cell death analyses were performed as previously described [15]. Attached and floating cells were collected. For cell cycle analysis they were stained with Nicoletti buffer (50 µg/µL propidium iodide (PI), 0.1% sodium citrate and 0.1% Triton X-100). To determine the numbers of apoptotic and necrotic cells, cells were incubated with Annexin V-FITC (Immunotools, Friesoythe, Germany) in Annexin V binding buffer and propidium iodide (PI) at 2 μg/mL. Flow cytometry analysis was performed using Miltenyi MACSQuant^®^ Analyzer (Milteny Biotec GmbH, Bergisch Gladbach, Germany) and MACSQuantify Software (Milteny Biotec GmbH, Bergisch Gladbach, Germany).

For the EdU pulse label experiment cells were cultured on six-well plates. Cells were treated for indicated time intervals with HDACi or DMSO as a control. During the last 2 h, cells were labeled with 10 µM EdU. Fixation and further steps were performed as described by the manufacturer (Click-Edu Flow Kit 647, Sigma Aldrich, St. Louis, MO, USA). Detection was performed using a Miltenyi MACSQuant^®^ Analyzer (Milteny Biotec GmbH, Bergisch Gladbach, Germany). 

To determine the number of cells in M phase double staining of phosphorylated histone H3 (Serin 10) and PI was performed. After HDACi treatment cells were detached and harvested. Fixation was done with 4% Formaldehyde in PBS. Cells were permeabilized for 5 min with 0.5% Triton X-100 in PBS. After washing, cells were incubated in antibody solution with pH3 antibody (1:1600, #53348, Cell Signaling Technology (CST), Danvers, MA, USA) and 0.5% BSA in PBS for 1 h. After washing, secondary antibody (1:500, Alexa-488-anti-rabbit, #A-11034, Thermo Fisher Scientific, Waltham MA, USA) was added in 0.5% BSA in PBS for 30 min. After the final wash cells were resupended in 500 μL PBS with 0.2% PI. Fluorescence was detected with MACSQuant^®^ Analyzer (Milteny Biotec GmbH, Bergisch Gladbach, Germany).

### 2.3. Histone Extraction

Histones were acid-extracted as described by Shechter et al. [36]. One microgram of each sample was used for western blot analysis with 15% SDS-PAGE gels as described in the next section.

### 2.4. Protein Expression Analysis

Immunoblot analysis was performed with whole cell extracts. Cells were lysed in radioimmunoprecipitation assay (RIPA)-buffer (150 mM NaCl, 1% Triton X-100, 0.5% deoxycholate, 1% Nonidet P-40, 0.1% SDS, 1 mM EDTA, 50 mM TRIS, pH 7.6), containing a protease inhibitor cocktail (10 µL/mL, Sigma-Aldrich, St. Louis, MO, USA). BCA assay (Thermo Fisher Scientific, Waltham MA, USA) was used to determine protein concentration. Twenty micrograms of each protein lysate were used for SDS-PAGE followed by transfer onto PVDF membrane (Merck Millipore, Schwalbach, Germany) by electroblotting. Membranes were blocked with skimmed milk solution prepared in TBST (150 mM NaCl, 10 mM TRIS, pH 7.4 and 0.1% Tween-20) and probed with respective primary antibodies (Table S1). After washing, secondary horseradish peroxidase-conjugated-antibodies were probed (Table S1). Targets were visualized by SuperSignal™ West Femto (Thermo Scientific, Rockford, IL, USA) and WesternBright Quantum kit (Biozym, Hessisch Oldendorf, Germany).

### 2.5. Database Analysis, Software, and Statistics

Mutations and copy number changes (CNA) in UCC were queried using the Cancer Cell Line Encyclopedia database (https://portals.broadinstitute.org/ccle, accessed on 10 December, 2020). Genetic alterations in patient tissues were queried using TCGA data for the BLCA dataset (*n* = 413, [37]) via the cBioPortal database (https://www.cbioportal.org/, accessed on 10 December 2020) [38,39]. 

IC_50_ concentrations were calculated by means of the AAT-Bioquest^®^ online calculator tool. To determine drug synergy for combined treatment with Romidepsin and MK-1775 the compounds were applied to the cells in fixed dose ratios. Individual dose ratios were chosen based on the IC_50_ of the individual drugs for each cell line. Six different combinations of concentrations were applied and then analyzed by the Chou-Talalay method using the CompuSyn software [40].

GraphPad Prism version 8 (GraphPad Software, San Diego, CA, USA) was used to generate graphs and calculate p-values according to Mann–Whitney. 

## 3. Results

### 3.1. Cyclin/CDK Complexes and G2/M Checkpoint Control Genes Are Rarely Mutated in UC Tissues and Cell Lines

**Figure 1:**
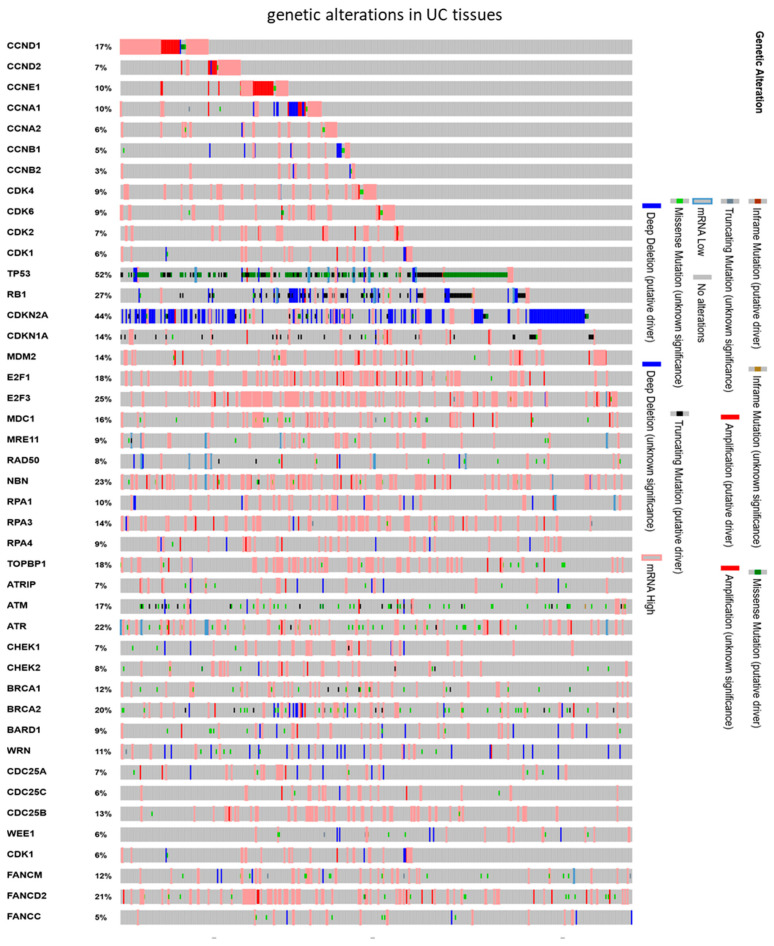
Genetic alterations of cell cycle and checkpoint control genes in UC tissues. Public TCGA data was queried for the BLCA set (*n* = 413; [37]) by means of the oncoprint tool provided by the cBioPortal database.

In previous work we observed accumulation of HDACi-treated UC cells in the G2/M phase [15]. In this study, we sought to investigate the underlying mechanisms to identify new partners for combined treatment of UC cells. Thus, we examined public data for genetic inactivation of possibly involved genes. A query of public TCGA data for genetic alterations in 413 UC tissues revealed mainly well-known drivers of UC, like *TP53* mutations or *RB1* and *CDKN2A* deletions (Figure 1) [41]. Other genes like E2F transcription factors or DNA damage signaling components like *ATM* and *ATR* were affected by missense mutation of unknown significance or rather overexpressed due to amplification. 

**Figure 2:**
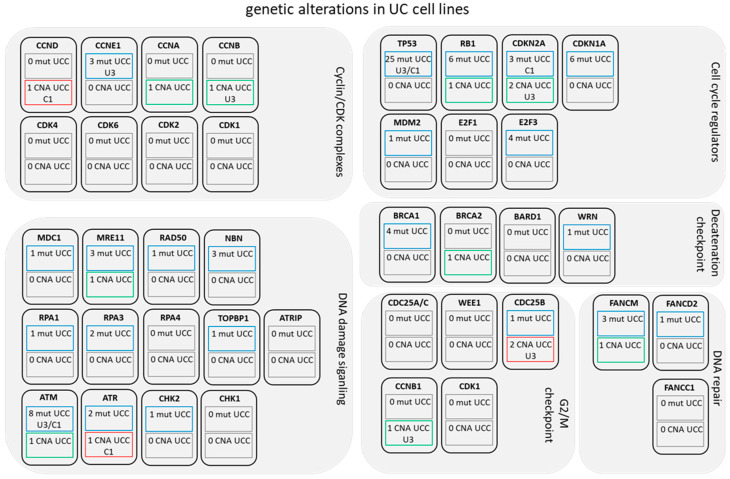
Alterations in cell cycle and checkpoint control genes in UC cell lines by genetic changes. CCLE data was queried via the Broad Institute database for 31 urinary tract cancer cell lines for genetic mutations. Queried genes are displayed as boxes arranged according to their regulatory function in cell cycle control and DNA damage response. Upper inserted blue lined boxes mark genes with known mutations in the number of UC cell lines. If the two UC cell lines VM-CUB1 and UM-UC-3 were among the mutated lines, they are listed specifically below as “C1” and “U3”. Grey lined boxes indicate no mutation in the 31 cell lines. The second box below likewise informs about copy number alterations (CNA). Green lined boxes indicate deletions, red lined boxes indicate amplifications.

A similar analysis was performed for urinary tract cancer cell lines and revealed comparable frequencies of genetic changes (Figure 2). The Cancer Cell Line Encyclopedia (CCLE) comprises 31 cell lines (listed in Table S2) with mutational data (compared to 413 tissues), thus considering the frequency of detected changes in the 413 tissues only few cell lines harbored mutations or copy number alterations (CNA). Again, the most frequently altered genes were *TP53*, *RB1*, and *CDKN2A*. Detailed information on the type of mutations and copy number changes for the affected genes in UCCs is summarized in Tables S2 and S3. Importantly, only rare mutations affected G2/M molecules in UCC suggesting this checkpoint to be functional. The two UC cell lines we treated with compounds in the following, VM-CUB1 and UM-UC-3, had generally only few mutations and CNAs of the relevant genes. Of the 42 queried genes (summarized in Figure 2), only three (*TP53, CDKN2A, ATM*) were genetically altered in both cell lines. Both cell lines are hypertriploid with mutant *TP53* and wild-type *RB1*. UM-UC-3 carries homozygous deletions of *CDKN2A*, which is mutated in VM-CUB1.

Taken together, cellular response of UC cells to HDACi differing from other entities may not only originate from cancer-specific genetic changes. They may also be elicited by UC-specific functions of different HDAC isoenzymes in the regulation of expression of cell cycle and checkpoint control genes. Therefore, we next characterized the effect of class I-HDACi with different isoenzyme specificities on the cellular response of UC cells and protein expression changes. 

### 3.2. Romidepsin Induces Cell Death More Strongly Than Entinostat and RGFP966

To analyze cellular responses of UC cell lines (UCC) to the class I-specific HDACi Romidepsin, Entinostat, and RGP966, IC_50_ doses were determined by MTT viability assay for the UCC VM-CUB1 and UM-UC-3 and the normal control cell line HBLAK [33]. These analyses have been published previously for Romidepsin and Entinostat [15] and were performed for RGFP966 here. IC_50_ doses for Romidespin were in the low nanomolar range, for Entinostat in the low micromolar range and above 20 µM for RGFP966 doses (Table S4). 

Efficacy of the treatment at the molecular level was validated by analysis of histone acetylation changes 24 and 48 h after treatment with IC_50_ doses of each inhibitor. To this end, histone extracts were analyzed by western blotting for acetylated histone H3 as well as total histone H3 and H4 (Figure S1). Histone acetylation levels were strongly increased after treatment with each inhibitor. 

**Figure 3:**
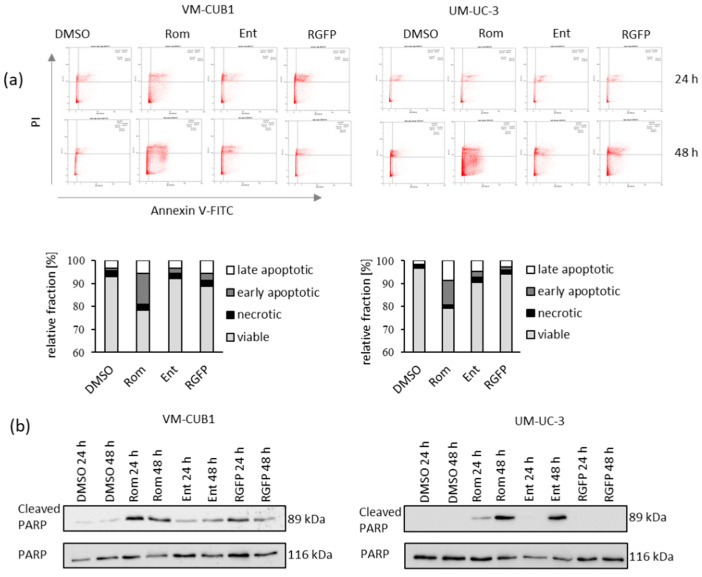
Induction of cell death in UCC after treatment with class I-specific HDACi. (**a**) Induction of cell death was measured by Annexin V/PI staining and flow cytometry 24 and 48 h after treatment with indicated HDACi Romidepsin (Rom), Entinostat (Ent) or RGFP966 (RGFP) compared to DMSO control cells. Cells in the lower left quadrant are viable, in the lower right early apoptotic, in the upper right late apoptotic and in the upper left necrotic. Example raw data of one representative experiment is visualized. Plots for 48 h treatment are additionally displayed enlarged in Figure S2. The means for percentages of cells in the respective quadrants after 48 h from three independent experiments are summarized in bar graphs. (**b**) PARP cleavage was determined by western blot analysis after treatment for 24 and 48 h with indicated HDACi. Total PARP protein was detected as a loading control.

Next, we measured induction of cell death by HDACi in UCC after treatment with IC_50_ doses by staining of Annexin V and propidium iodide (PI) and flow cytometry after 24 and 48 h (Figure 3a). Romidepsin, which targets mainly HDAC1 and 2, induced cell death most strongly which occurred by a mixture of apoptosis and necrosis. The two compounds inhibiting HDAC3 induced significantly less cell death. Accordingly, PARP cleavage in UCC was induced most strongly and earliest by Romidepsin. Of note, UM-UC-3 cells responded with a delayed kinetic compared to VM-CUB1 (Figure 3b). 

### 3.3. Inhibition of HDAC1 and HDAC2, But Not of HDAC3 Results in Accumulation of Cells in G2/M Phase

**Figure 4:**
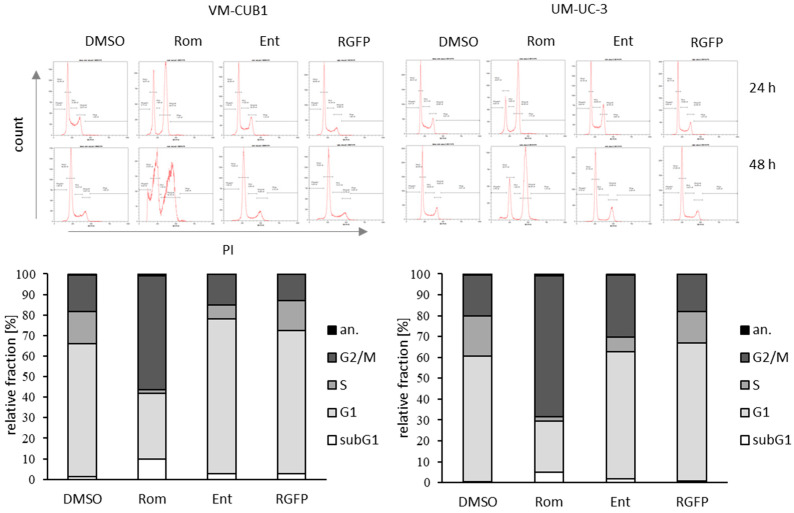
Cell cycle analysis of UCC after treatment with class I-specific HDACi. Cell cycle alterations were monitored by PI staining and flow cytometry 24 and 48 h after treatment with the indicated HDACi. Respective cell cycle phases were gated and percentage of cells within gates calculated. Example raw data of one representative experiment is visualized. The means for percentage of cells in the respective cell cycle phase after 48 h from three independent experiments are summarized in bar graphs.

In cell cycle analyses of UCC cells by flow cytometry after 24 and 48 h we observed severe alterations following treatment with Romidepsin, but not with the two compounds that target HDAC3. Only treatment with Romidepsin resulted in strong accumulation of cells in the G2/M phase (Figure 4). Furthermore, we noticed a small increase of aneuploid cells (black fraction in bar graph). Entinostat treated VM-CUB1 cells displayed increased numbers of cells in G1. The number of cells in subG1 pointing at cell death induction concurred with previous cell death analysis confirming most prominent effects by Romidepsin (Figure 3). RGFP966, which exclusively inhibits HDAC3, had almost no effect on the cell cycle.

**Figure 5:**
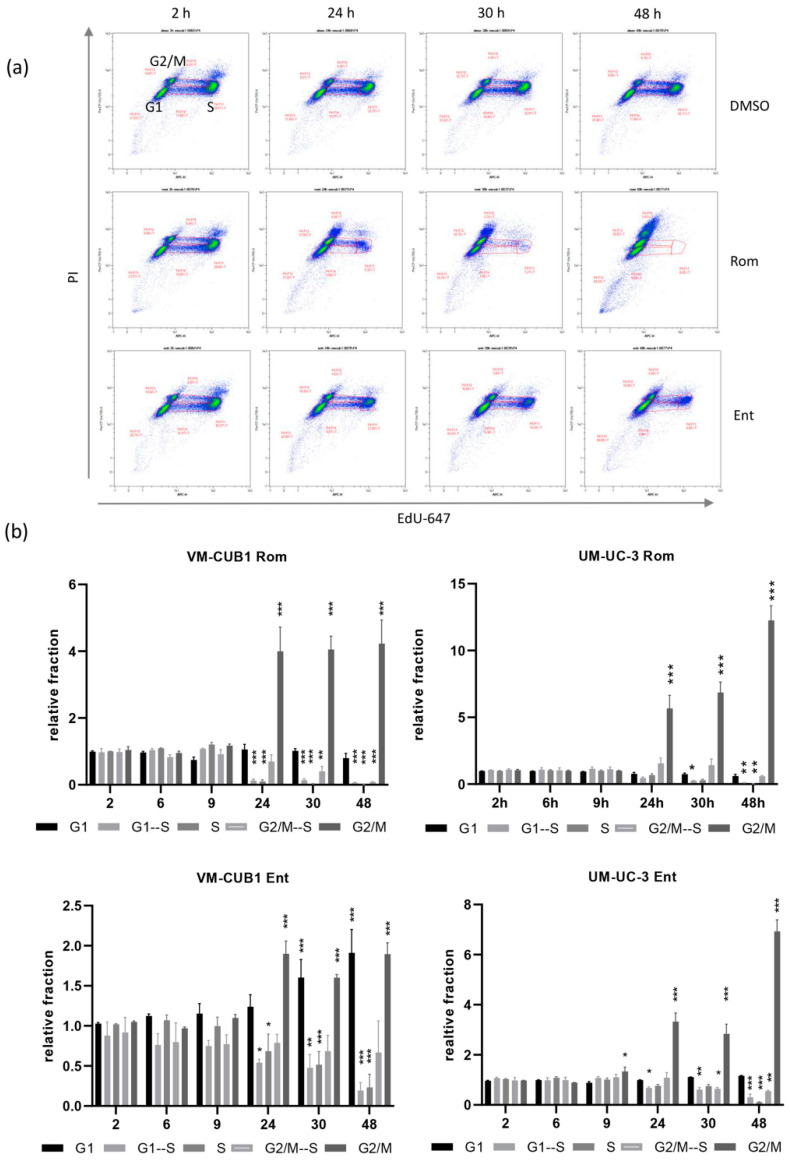
EdU label experiment with UCC after treatment with class I-specific HDACi. (**a**) Raw data plots for VM-CUB1 cells displaying Alexa-647 labeled EdU and PI after indicated treatments and time points. Different cell populations reflecting EdU-labeled or unlabeled cells are displayed in respective cell cycle phases. Example raw data of one representative experiment is visualized. (**b**) The means for percentages of cells in cell populations over time (indicated in hours) of three independent experiments were summarized as bar graphs. Results were normalized to DSMO control. *** *p* ≤ 0.001, ** *p* ≤ 0.01, * *p* ≤ 0.05.

To follow UC cells through the cell cycle in a more detailed manner, we performed an EdU pulse label experiment that confirmed significant accumulation of Romidepsin-treated cells in G2/M and reduced numbers of cells in S phase as well as in the transition from G1 to S and S to G2/M (Figure 5a,b). Entinostat treatment of VM-CUB1 generated a mixed pattern with cells accumulated in G1 and G2/M. Since RGFP966 treatment did not result in significant cell cycle changes (Figure 3), pulse labeling was not performed for the HDAC3 inhibitor. 

### 3.4. Romidepsin Treatment Results in Cell Cycle Arrest in G2 and Loss of M-Phase Cells

**Figure 6:**
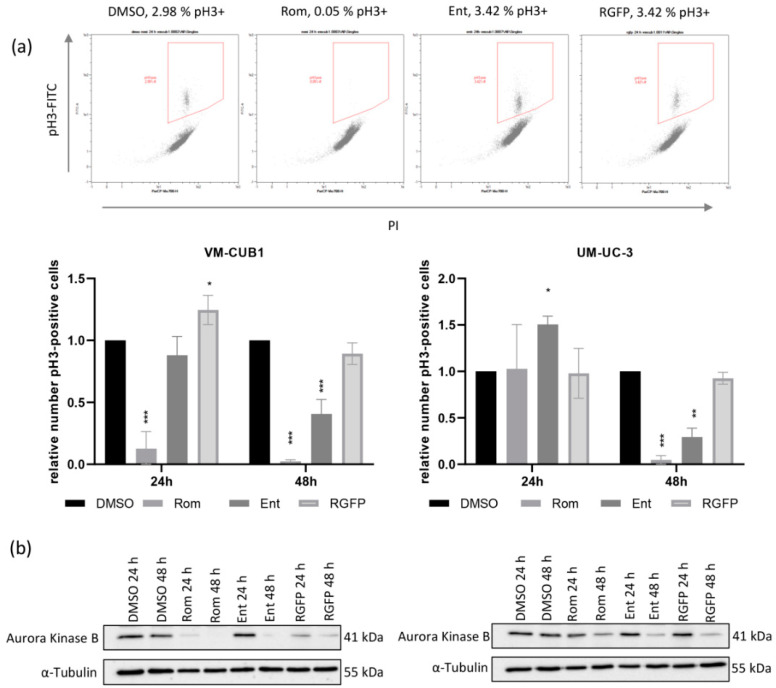
Phosphorylation of histone H3 is abolished by inhibition of HDAC1 and HDAC2 through Romidepsin treatment. (**a**) UC cells treated with indicated HDACi were stained for phosphorylated H3 and with PI and analyzed by flow cytometry after 24 h and 48 h. Cells double positive for PI and pH3 were gated as cells in M phase. Example raw data of one representative experiment for VM-CUB1 24 h after indicated treatment is visualized. Numbers of cells positively stained for phosphorylated H3 (pH3+) are given above the plots. Further example raw data is shown enlarged in Figures S3 and S4. Means of independent experiments are displayed in bar graphs. Relative number of double positive cells after normalization to DMSO controls is given. *** *p* ≤ 0.001, ** *p* ≤ 0.01, * *p* ≤ 0.05. (**b**) Protein expression of AURKB was determined by western blot analysis in VM-CUB1 and UM-UC-3 cells 24 h and 48 h after treatment with the indicated HDACi. α-Tubulin was stained as a loading control.

To further differentiate between cells in G2 and M phase, we compared staining for phosphorylated histone H3 (pH3) against PI by flow cytometry after 24 h and 48 h. H3 becomes phosphorylated at Serin 10 by Aurora Kinase B (AURKB) at the entry of M phase [42]. We discovered that the number of UC cells positively stained for pH3 arriving in M-phase was strongly reduced following inhibition of HDAC1 and HDAC2 by Romidepsin (Figure 6a, Figures S3 and S4). After 48 h no cells were detectable in M phase anymore indicating that Romidepsin induced cell cycle arrest in G2 phase. The number of cells in M-phase was only partially reduced by Entinostat after 48 h. This effect presumably originates from inhibition of HDAC1 rather than HDAC3, since the HDAC3-specific inhibitor RGFP966 did not significantly affect transition towards M phase. As observed earlier, UM-UC-3 cells displayed a delayed response with no changes after 24 h, but also complete arrest in G2 after 48 h. Taken together, inhibition of HDAC1 and HDAC2, but not of HDAC3 resulted in complete arrest in G2. 

To elucidate the mechanism underlying changes in H3 phosphorylation, we examined expression of AURKB protein. Concurringly, AURKB was completely lost by Romidepsin treatment in VM-CUB1 cells already after 24 h (Figure 6b). Entinostat also reduced AURKB expression after 48 h. UM-UC-3 cells responded comparably, but less strongly and with a delay after 48 h. In summary, the level of AURKB expression correlated with the level of H3 phosphorylation and a complete loss of AURKB activity contributed to loss of histone H3 phosphorylation. 

### 3.5. Inhibition of HDAC1 and HDAC2 Induces DNA Damage and Impairs DNA Damage Checkpoint Signaling

**Figure 7:**
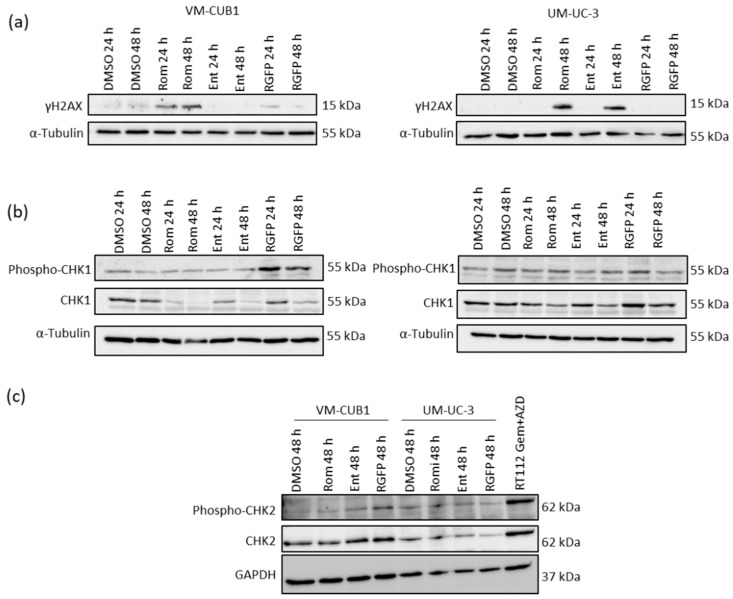
Analysis of DNA damage and according checkpoint signaling after treatment with HDACi. (**a**) Phosphorylation of γH2AX at Serin 139 was detected by western blotting after treatment with indicated HDACi for 24 h and 48 h. (**b**) Phosphorylated and total CHK1 protein was detected by western blotting after treatment with the indicated HDACi for 24 h and 48 h. α-Tubulin was stained as additional loading control. (**c**) Likewise, phosphorylated and total CHK2 was detected. RT112 UCC cells treated with Gemcitabine and AZD7762 (Gem+AZD) were used as a positive control for CHK2 activation [45]. α-Tubulin and GAPDH were stained as additional loading control.

Since we had observed in previous work that HDACi can induce DNA damage [15,18,43], we detected phosphorylated γH2AX as DNA damage marker in UCC treated with the three class I–specific HDACi. In VM-CUB1 cells we detected γH2AX only after treatment with Romidepsin. In UM-UC-3 cells, it became detectable 48 h after treatment with compounds inhibiting HDAC1 and HDAC2, but not with the HDAC3-specific inhibitor (Figure 7a). Since γH2AX is phosphorylated during DNA damage response signaling [44], we investigated the activity and expression of checkpoint kinases (CHK) after HDACi treatment. We discovered that CHK1 was not activated by phosphorylation after treatment with Romidepsin or Entinostat and was in fact downregulated (Figure 7b). Surprisingly, CHK1 phosphorylation was detectable after treatment with the HDAC3 inhibitor RGFP966 in VM-CUB1 cells even though γH2AX was not significantly phosphorylated. Thus, checkpoint signaling in S and G2 phase was impaired after inhibition of HDAC1 and HDAC2, but not of HDAC3. CHK2 kinase was not significantly altered by treatment with any HDACi.

In summary, we observed that inhibition of HDAC1 and HDAC2 by Romidepsin induced DNA damage, but did not activate checkpoint kinase signaling. Still, treated cells accumulated in G2 and could not transit into M-phase after 48 h. We therefore hypothesized that G2/M signaling might mediate cell cycle arrest to limit the anti-neoplastic activity of HDACi. This was raising the possibility that inhibiting additionally the G2/M checkpoint pharmacologically, by combined treatment with Romidepsin and the WEE1 inhibitor MK-1775, might push UCC into mitotic catastrophe and enhance cell death. 

### 3.6. Combined Treatment with Romidepsin and MK-1775 Only Marginally Increases the Number of Cells Entering Mitosis and Subsequent Cell Death

**Figure 8:**
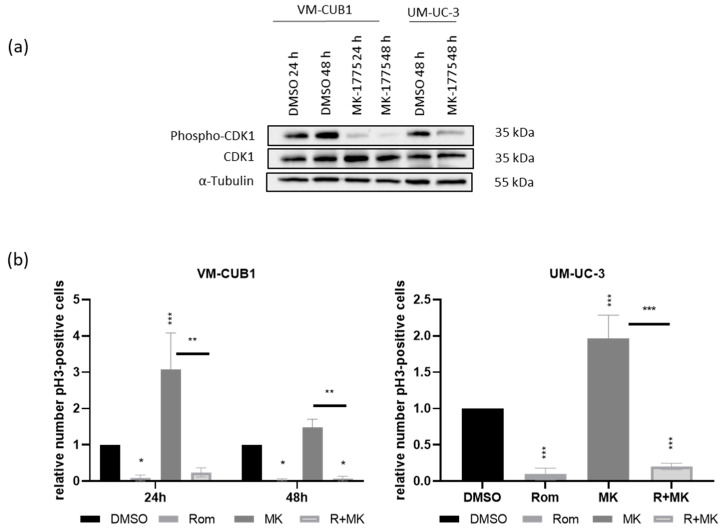
Molecular analysis of MK-1775 treatment efficacy. (**a**) Phosphorylation of CDK1 was detected by western blotting after treatment with the WEE1 inhibitor MK-1775 (MK) at indicated time points. Since UM-UC-3 cells repeatedly displayed a delayed response the 24 h time point was omitted. Total CDK1 protein and α-Tubulin were stained as loading controls. (**b**) UC cells treated with MK, Rom, or the combination (R+MK) were stained for H3 and PI and subsequently analyzed by flow cytometry after 24 h and 48 h. Cells that were double positive for PI and pH3 were gated as cells in M phase. Means of independent experiments are displayed in bar graphs. Relative number of double positive cells after normalization to DMSO controls is given. *** *p* ≤ 0.001, ** *p* ≤ 0.01, * *p* ≤ 0.05.

The G2/M checkpoint prevents cells with damaged DNA from progressing to mitosis. DNA damage can activate CHK1 via phosphorylation by ATM and ATR. CHK1 can then inactivate CDC25C phosphatase and activate WEE1 kinase by phosphorylation. Both proteins regulate the CDK1/CyclinB1 (CCNB1) complex that promotes transition to mitosis. CDK1 becomes activated by dephosphorylation through CDC25C and is inactivated by phosphorylation through WEE1, which results in G2 arrest [46]. Accordingly, pharmacological inhibition of WEE1 can activate CDK1/CCNB1 and allow progression into mitosis despite DNA damage signaling. MK-1775 (AZD1775) is a specific inhibitor of WEE1 that is already evaluated in clinical trials [47]. Dose response analyses of MK-1775 for UCC VM-CUB1 and UM-UC-3 rendered similar IC_50_ concentrations in the very low micromolar range (Table S4). Interestingly, benign HBLAK control cells were even more sensitive to treatment with MK-1775. Western blot analysis of CDK1 phosphorylation demonstrated that MK-1775 indeed efficiently reduced phosphorylation of CDK1 (Figure 8a). 

Next, we investigated whether treatment with the checkpoint inhibitor alone would increase the number of cells transiting into M-phase. As expected, flow cytometry analysis of pH3 demonstrated increased numbers of positive cells after MK-1775 treatment indicating that UCC progressed towards M phase (Figure 8b). However, combined treatment with the cell line-specific IC_50_ dosage of the HDACi and the checkpoint inhibitor increased the number of cells progressing into M phase only slightly. 

**Figure 9:**
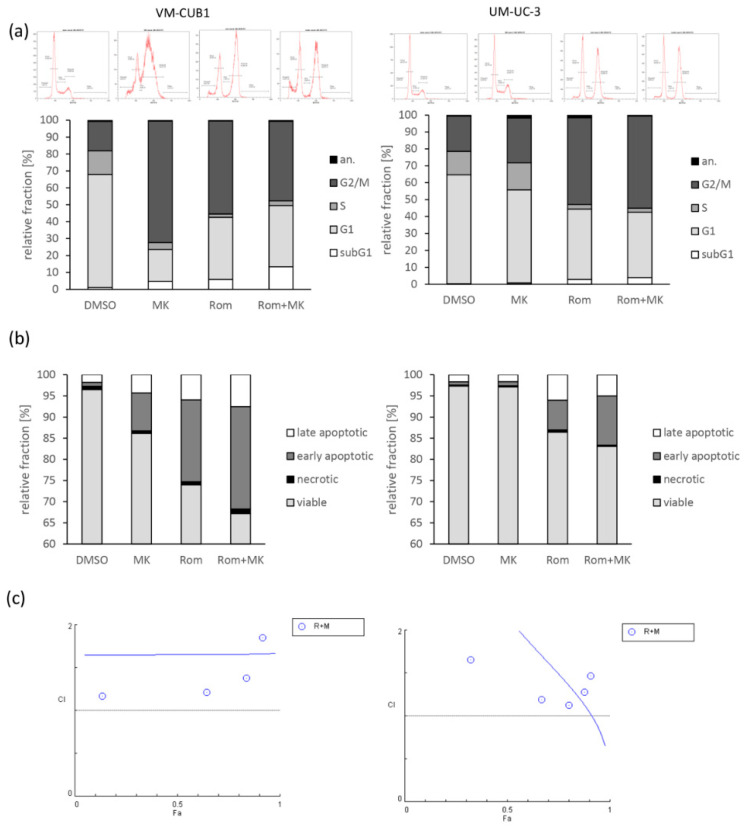
Effects of combined treatment on cell cycle and cell death induction. (**a**) Cell cycle alterations were monitored by PI staining and flow cytometry 24 h and 48 h after single or combined treatment (DMSO, MK, Rom, Rom + MK). Example raw data from a representative experiment is displayed. Means of independent experiments for percentages of cells in the respective gates after 48 h are illustrated in bar graphs. (**b**) Induction of cell death was measured by Annexin V/PI staining and flow cytometry 24 h and 48 h after the indicated combined or single treatment. Means of independent experiments for percentages of cells in the respective quadrants after 48 h are illustrated in bar graphs. (**c**) Combination index (CI) plots for the combination of Romidepsin and MK-1775 (R + M). Cell viability was measured at six constant dose ratios by MTT assay after 72 h treatment. Actual applied combination dosages are given in Table S4. Combination index versus the number of dead cells (Fraction affected: Fa) were calculated and CI plots were generated using CompuSyn software. CI < 1 indicates synergism, CI = 1 additive effects and CI > 1 antagonism.

Cell cycle analysis of UCC treated with the combination demonstrated only small additional changes compared to single treatments (Figure 9a). Likewise, analysis of cell death induction after single or combined treatment revealed only small increases after combination treatment when compared to treatment with Romidepsin only (Figure 9b). PARP cleavage was also not further increased by combined treatment (Figure S5). Consequently, analysis of viability assays with combination dosage ranging between 0 and 2-fold of the cell line-specific IC_50_ concentrations (actual doses are given in Table S4) by the Chou-Talalay method showed no synergistic effect for the combined treatment (Figure 9c) [40]. Taken together, combined treatment with the HDACi Romidepsin and the G2/M checkpoint inhibitor MK-1775 did not significantly increase the antineoplastic effect on UCC. Normal HBLAK control cells as well were almost unaffected by combined treatment (Figure S6, Table S4). To understand which mechanisms prevent synergistic effects we further investigated the involved key players of the G2/M checkpoint in UC cells.

### 3.7. Inhibition of HDAC1 and HDAC2 Impairs G2/M Checkpoint Control by Downregulation of Key Genes 

**Figure 10:**
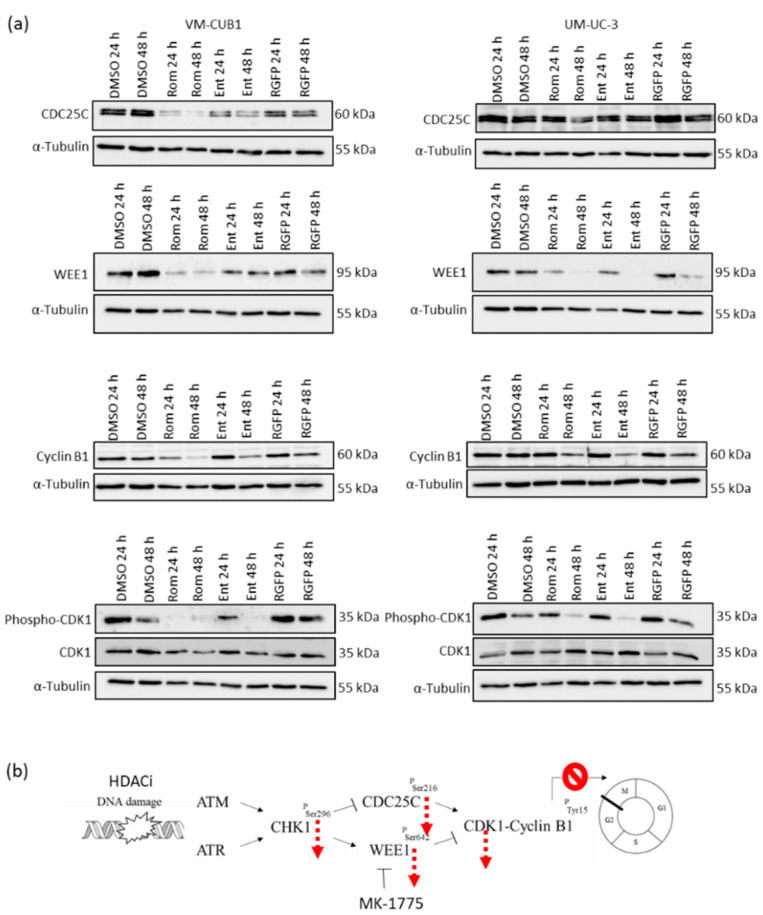
Protein expression of key regulators of the G2/M checkpoint after HDACi treatment. (**a**) Protein expression was examined by western blot analysis for CDC25C, WEE1, CCNB1 and phosphorylation of CDK1 after treatment with the indicated HDACi at the indicated timepoints. α-Tubulin and total CDK1 were detected as a loading control. (**b**) The observed changes are assembled into a model of effects of HDACi on G2/M checkpoint regulation that explains why combined treatment with Romidepsin and MK-1775 did not result in synergistic anti-neoplastic effects.

Since MK-1775 action depends on WEE1 and on the phosphatase CDC25C, the effects of Romidepsin and Entinostat on these regulators of the G2/M checkpoint were analyzed by western blotting. Indeed, both HDACi downregulated CDC25C, particularly in VM-CUB1 cells (Figure 10a). Moreover, WEE1 was even more downregulated by both HDACi. Thus, the target of MK-1775 was already downregulated by inhibition of HDAC1 and HDAC2. In addition, the HDACi reduced expression of the cyclin CCNB1 and phosphorylation of CDK1. Reduced phosphorylation of CDK1 might promote cell cycle progression, but expression of CCNB1 would be needed for an active CDK1/CCNB1 mitosis promoting complex. Taken together, these observations suggest a model whereby inhibition of HDAC1 and HDAC2 downregulates several essential G2/M checkpoint regulators, so that even pharmacological inhibition of WEE1 cannot push cells into mitotic catastrophe and therefore does not further increase the anti-neoplastic efficacy of the HDACi treatment (Figure 10b).

## 4. Discussion

In previous studies we identified specific inhibition of class I HDACs, especially of HDACs 1, 2, and 3 as a more efficacious therapeutic approach for UC than application of pan-HDAC inhibitors like Vorinostat [11,15,18]. We also demonstrated previously that class I-specific inhibitors like Romidepsin and Givinostat efficiently affect UCC, but spared various normal control cell lines [15]. With the present study, we can further refine candidates for targeted therapy since our data demonstrate that HDAC1 and HDAC2 are better targets than HDAC3 and that inhibition of HDAC3 may even be counterproductive. HDAC1 and 2 are components of different multiprotein complexes than HDAC3 [26,27]. Accordingly, different functions for HDAC1/2 versus HDAC3 have been reported e.g., in DNA damage response [28,48,49]. 

We also followed up on the unexpected observation that HDACi inhibiting HDAC1/2, but not HDAC3 lead to accumulation of UC cells in G2/M phase, whereas HDACi treatment of many other cancer cell entities—solid and hematopoietic—usually results in G1 arrest [19,20,21,22,23,24,25]. The in silico analyses on mutations and copy number alterations in UC presented here make it unlikely that these differences between cancer cell entities originate from differences in genetic changes in cell cycle and checkpoint control genes. While cell cycle regulators in bladder cancers may be more regularly mutated than in some other cancer types [50], most alterations concern *TP53*, *RB1*, and *CDKN2A*, which are also frequently mutated in other cancer types—e.g., lung, colorectal, and brain cancers—and are known to contribute to deficient G1 checkpoint control in UC [51]. However, colon, and lung cancer cells still respond to Romidepsin with cell cycle arrest in G1 and not G2/M [21,23]. The additional search presented here did not reveal significantly prevalent further mutations or copy number alterations in bladder cancer tissues or cell lines that would explain why UCC should react differently from other cancer types. Conceivably, however, G1 checkpoint deficient cells may rely more strongly on functional G2/M checkpoints (see below). Accordingly, no additional genetic changes in G2/M regulators genes are detected in bladder cancer tissues and cell lines.

Thus, we concluded that differences in cell cycle arrest between UC cells and other cancer cell types after treatment with HDACi do not originate from cancer-specific genetic changes, but may rather be elicited by UC-specific functions of HDAC isoenzymes in the regulation of cell cycle and checkpoint control genes. In particular, we observed a strong effect of HDAC1/2 inhibition on expression of genes encoding DNA damage and G2/M checkpoint regulators like *CHK1*, *AURKB*, *CCNB1*, *WEE1*, and *CDC25C* which may partly account for the different response of UC cells.

Usually, checkpoint activation via ATM and CHK2 results in degradation of CDC25A and inhibition of CDK2 thereby inhibiting transition from G1 into S which requires CCNE/CDK2 activity [52]. However, cells damaged in late G1 may still progress into S-phase leading to accumulation in G2. This escape from checkpoint activation occurs more easily in cells with a high level of active CCNE/CDK2 and is favored by deficiencies in G1 checkpoint control like those prevalent in bladder cancer. We reported previously that Romidepsin induced high levels of CCNE in VM-CUB1 and UM-UC-3 cells which may further help the cells to progress in cell cycle [15]. Additionally, both cell lines carry *ATM* mutations, and we did not observe activation of CHK2. These relations might thus explain why UC cells were not arrested in G1 by HDACi, but rather proceeded to G2/M. 

By further in depth analysis of cell cycle changes by EdU labeling and detection of pH3 as a marker for mitosis entry, we could clarify that Romidepsin treatment resulted in accumulation in G2. Complete abolishment of M phase cells was achieved by Romidepsin treatment, while Entinostat only reduced the number of M phase cells. Along with pH3, expression of the responsible kinase AURKB was downregulated by HDACi targeting HDAC1 or HDAC2. Even though RGFP966 targeting HDAC3 also reduced AURKB expression to some extent, it did not significantly impede progression into mitosis. Obviously, residual AURKB expression was sufficient to phosphorylate histone H3. Downregulation of Aurora kinases by HDACi has been reported rarely yet, e.g., for lung cancer cells [53]. 

Accumulation in G2 may generally be elicited by checkpoint activation following DNA damage signaling. Indeed, phosphorylated γH2AX as a marker for DNA damage became detectable after treatment with Romidepsin in both cell lines and in UM-UC-3 cells also by Entinostat. Surprisingly, DNA damage did not result in activation of checkpoint kinases and rather a downregulation of CHK1 protein expression was observed. Interestingly, CHK1 downregulation by HDACi was also described for lung cancer and glioblastoma cells that too accumulated in G2 after HDACi treatment [54,55]. In contrast, Goder et al. reported that treatment of HCT116 cells with Entinostat arrested the cells in G1 phase and prevented activation of checkpoint kinases through phosphorylation, but not their expression [56]. Downregulation of CHK1 expression by HDACi thus appears to be cell type dependent and occurred in UC cells after inhibition of HDAC1 and HDAC2, but not of HDAC3. Induction of DNA damage in cells with abrogated checkpoints may lead to progression into mitosis, resulting in mitotic catastrophe and consequently cell death [52]. Thus, a combination treatment of class I HDACi and MK-1775 targeting G2/M checkpoint signaling appeared a reasonable approach to drive UC cells into mitotic catastrophe to enhance cell death [57]. Synergism between HDACi and MK-1775 has been described for neuroblastoma and acute myeloid leukemia cells [58,59]. However, even though MK-1775 treatment alone increased the number of UC cells in M phase, combined treatment with Romidepsin did not lead to more M-phase cells compared to single treatment. Accordingly, we did not observe synergism and induction of cell death by the combined treatment was rather additive. This failure is explained by the broad expression changes of G2/M regulators by HDACi as follows. 

While CHK1 and CHK2 share substrate homology, CHK1 is believed to be the main executory kinase [60]. Accordingly, we observed before that UCC were more sensitive to siRNA-mediated knockdown of CHK1 than CHK2 and interference with CHK1 resulted in more pronounced sensitization towards gemcitabine treatment [45]. CHK1 phosphorylates phosphatases of the CDC25 family that further propagate checkpoint signaling, leading to intra-S phase and G2/M phase arrest by inactivating the CCNB1/CDK1 complex. Phosphorylation of CDK1 by WEE1 kinase inhibits its activity during G2, whereas dephosphorylation by CDC25 family members results in activation of CDK1 during early mitosis. CDK1 interacts with its activator CCNB1 to form “the mitosis-promoting factor” heterodimer [52], which is required until the metaphase. Progression into anaphase is dependent on its sudden destruction by the anaphase-promoting complex (APC) [61]. Thus, the activity of CDK1 is regulated at several levels: (1) at the transcriptional level for CCNB1 and CDK1, (2) via its phosphorylation state by kinases and phosphatases as a downstream consequence of CHK1 signaling and (3) through degradation of CCNB1 [52]. We found CDC25C and WEE1, the two key factors regulating activity of the mitosis promoting CCNB1/CDK1 complex to be downregulated by HDACi targeting HDAC1/2, but not HDAC3. Inhibitory phosphorylation of CDK1 was reduced by HDACi, but expression of its complex activator CCNB1 was also downregulated which may prevent progression in the cell cycle. In addition, lack of AURKB, with its various functions in cell cycle, may also prevent progression of HDACi treated cells into mitosis and limit the effect of the combination treatment [62]. 

Loss of CHK1 and WEE1 expression was also reported for HDACi treated glioblastoma cells [55]. Again, this appears to be tissue-specific since Entinostat treated HCT116 cells also responded with downregulation of WEE1 expression, but increased CCNB expression which allowed the cells to progress towards mitotic catastrophe. One important difference between UCC and HCT116 cells is that the latter are *TP53* wildtype. Nevertheless, MK-1775 was described as synergizing particularly in *TP53* mutant cells with other DNA damage inducing compounds [63]. Downregulation of WEE1 may further contribute to lacking CHK1 signaling since WEE1 is needed for ATR/CHK1 activation and WEE1 inhibition mediates inactivation of CHK1 [64]. As a consequence, loss of ATR, CHK1, and WEE1 can lead to excessive generation of ssDNA, leading to formation of DSB and cell death [63]. This relation may constitute one mechanism underlying the DNA damage observed following treatment with HDACi targeting HDAC1/2. 

## 5. Conclusions

Our study demonstrates that targeting of HDAC1 and HDAC2, but not of HDAC3, is a promising modern approach for treatment of UC. Romidepsin was the most efficient inhibitor to induce cell cycle alterations and cell death at low nanomolar doses. In contrast to many other cancer entities, this HDACi induced G2 cell cycle arrest. This cancer type-specific effect may be partly due to the deficient G1 checkpoint in UC, but our new data indicates that G2 arrest is elicited by downregulation of the DNA damage checkpoint kinase CHK1 and several components of the G2/M checkpoint needed for cell cycle progression. This downregulation also prevents the expected synergism with the G2/M checkpoint inhibitor MK-1775. Broad interference with cell cycle and checkpoint regulators as a consequence of HDAC1/2 inhibition may also constitute one mechanism underlying induction of DNA damage by HDACi. Thus, combinations of HDACi with compounds inhibiting DNA damage repair should be investigated for bladder cancer therapy in the future.

## Data Availability

The data presented in this study is comprehensively shown in this article and its supplementary files. The underlying unprocessed raw data is available upon request.

## Supplementary Materials

The following are available online at https://www.mdpi.com/2073-4425/12/2/260/s1, Figure S1: Histone acetylation changes after treatment with HDACi. Figure S2: PARP cleavage as a measure of cell death after treatment with Romidepsin and MK-1775. Figure S3: Dose–response curve and combination index (CI) plot for the combination of Romidepsin and MK-1775 (R+M) in HBLAK. Figure S4: Example flow cytometry raw data for induction of cell death after HDACi treatment. Figure S5: Example flow cytometry raw data for phosphorylated H3 in VM-CUB1 cells treated with different HDACi. Figure S6: Example flow cytometry raw data for phosphorylated H3 in UM-UC-3 cells treated with different HDACi. Table S1: Antibodies used in this study. Table S2: Mutations in queried genes in UCC according to CCLE database. Table S3: Copy number alterations of queried genes in UCC according to CCLE database. Table S4: IC_50_ doses for used pharmacological inhibitors and actual dosage for combination treatment with fixed dose ratios.

## References

[1] Ferlay J., Colombet M., Soerjomataram I., Mathers C., Parkin D.M., Pineros M., Znaor A., Bray F. (2019). Estimating the global cancer incidence and mortality in 2018: GLOBOCAN sources and methods. Int. J. Cancer.

[2] Kiemeney L.A., Witjes J.A., Verbeek A.L., Heijbroek R.P., Debruyne F.M. (1993). The clinical epidemiology of superficial bladder cancer. Dutch South-East Cooperative Urological Group. Br. J. Cancer.

[3] Niegisch G., Gerullis H., Lin S.W., Pavlova J., Gondos A., Rudolph A., Haas G., Hennies N., Kramer M.W. (2018). A Real-World Data Study to Evaluate Treatment Patterns, Clinical Characteristics and Survival Outcomes for First- and Second-Line Treatment in Locally Advanced and Metastatic Urothelial Cancer Patients in Germany. J. Cancer.

[4] Von der Maase H., Sengelov L., Roberts J.T., Ricci S., Dogliotti L., Oliver T., Moore M.J., Zimmermann A., Arning M. (2005). Long-term survival results of a randomized trial comparing gemcitabine plus cisplatin, with methotrexate, vinblastine, doxorubicin, plus cisplatin in patients with bladder cancer. J. Clin. Oncol..

[5] Galsky M.D., Arija J.A.A., Bamias A., Davis I.D., De Santis M., Kikuchi E., Garcia-Del-Muro X., De Giorgi U., Mencinger M., Izumi K. (2020). Atezolizumab with or without chemotherapy in metastatic urothelial cancer (IMvigor130): A multicentre, randomised, placebo-controlled phase 3 trial. Lancet.

[6] Powles T., Park S.H., Voog E., Caserta C., Valderrama B.P., Gurney H., Kalofonos H., Radulovic S., Demey W., Ullen A. (2020). Avelumab Maintenance Therapy for Advanced or Metastatic Urothelial Carcinoma. N. Engl. J. Med..

[7] Cancer Genome Atlas Research N. (2014). Comprehensive molecular characterization of urothelial bladder carcinoma. Nature.

[8] Casadevall D., Kilian A.Y., Bellmunt J. (2017). The prognostic role of epigenetic dysregulation in bladder cancer: A systematic review. Cancer Treat. Rev..

[9] Buckwalter J.M., Chan W., Shuman L., Wildermuth T., Ellis-Mohl J., Walter V., Warrick J.I., Wu X.R., Kaag M., Raman J.D. (2019). Characterization of Histone Deacetylase Expression Within In Vitro and In Vivo Bladder Cancer Model Systems. Int. J. Mol. Sci..

[10] Niegisch G., Knievel J., Koch A., Hader C., Fischer U., Albers P., Schulz W.A. (2013). Changes in histone deacetylase (HDAC) expression patterns and activity of HDAC inhibitors in urothelial cancers. Urol. Oncol..

[11] Pinkerneil M., Hoffmann M.J., Schulz W.A., Niegisch G. (2017). HDACs and HDAC Inhibitors in Urothelial Carcinoma—Perspectives for an Antineoplastic Treatment. Curr. Med. Chem..

[12] Suraweera A., O’Byrne K.J., Richard D.J. (2018). Combination Therapy With Histone Deacetylase Inhibitors (HDACi) for the Treatment of Cancer: Achieving the Full Therapeutic Potential of HDACi. Front. Oncol..

[13] Ververis K., Hiong A., Karagiannis T.C., Licciardi P.V. (2013). Histone deacetylase inhibitors (HDACIs): Multitargeted anticancer agents. Biologics.

[14] Witt O., Deubzer H.E., Milde T., Oehme I. (2009). HDAC family: What are the cancer relevant targets?. Cancer Lett..

[15] Pinkerneil M., Hoffmann M.J., Deenen R., Kohrer K., Arent T., Schulz W.A., Niegisch G. (2016). Inhibition of Class I Histone Deacetylases 1 and 2 Promotes Urothelial Carcinoma Cell Death by Various Mechanisms. Mol. Cancer Ther..

[16] Kaletsch A., Pinkerneil M., Hoffmann M.J., Jaguva Vasudevan A.A., Wang C., Hansen F.K., Wiek C., Hanenberg H., Gertzen C., Gohlke H. (2018). Effects of novel HDAC inhibitors on urothelial carcinoma cells. Clin. Epigenet..

[17] Lehmann M., Hoffmann M.J., Koch A., Ulrich S.M., Schulz W.A., Niegisch G. (2014). Histone deacetylase 8 is deregulated in urothelial cancer but not a target for efficient treatment. J. Exp. Clin. Cancer Res..

[18] Pinkerneil M., Hoffmann M.J., Kohlhof H., Schulz W.A., Niegisch G. (2016). Evaluation of the Therapeutic Potential of the Novel Isotype Specific HDAC Inhibitor 4SC-202 in Urothelial Carcinoma Cell Lines. Target Oncol..

[19] Bernhart E., Stuendl N., Kaltenegger H., Windpassinger C., Donohue N., Leithner A., Lohberger B. (2017). Histone deacetylase inhibitors vorinostat and panobinostat induce G1 cell cycle arrest and apoptosis in multidrug resistant sarcoma cell lines. Oncotarget.

[20] He B., Dai L., Zhang X., Chen D., Wu J., Feng X., Zhang Y., Xie H., Zhou L., Wu J. (2018). The HDAC Inhibitor Quisinostat (JNJ-26481585) Supresses Hepatocellular Carcinoma alone and Synergistically in Combination with Sorafenib by G0/G1 phase arrest and Apoptosis induction. Int. J. Biol. Sci..

[21] Karthik S., Sankar R., Varunkumar K., Ravikumar V. (2014). Romidepsin induces cell cycle arrest, apoptosis, histone hyperacetylation and reduces matrix metalloproteinases 2 and 9 expression in bortezomib sensitized non-small cell lung cancer cells. Biomed. Pharmacother..

[22] Li L.H., Zhang P.R., Cai P.Y., Li Z.C. (2016). Histone deacetylase inhibitor, Romidepsin (FK228) inhibits endometrial cancer cell growth through augmentation of p53-p21 pathway. Biomed. Pharmacother..

[23] Shi Y., Fu Y., Zhang X., Zhao G., Yao Y., Guo Y., Ma G., Bai S., Li H. (2020). Romidepsin (FK228) regulates the expression of the immune checkpoint ligand PD-L1 and suppresses cellular immune functions in colon cancer. Cancer Immunol. Immunother..

[24] Sikandar S., Dizon D., Shen X., Li Z., Besterman J., Lipkin S.M. (2010). The class I HDAC inhibitor MGCD0103 induces cell cycle arrest and apoptosis in colon cancer initiating cells by upregulating Dickkopf-1 and non-canonical Wnt signaling. Oncotarget.

[25] Xue K., Gu J.J., Zhang Q., Mavis C., Hernandez-Ilizaliturri F.J., Czuczman M.S., Guo Y. (2016). Vorinostat, a histone deacetylase (HDAC) inhibitor, promotes cell cycle arrest and re-sensitizes rituximab- and chemo-resistant lymphoma cells to chemotherapy agents. J. Cancer Res. Clin. Oncol..

[26] Codina A., Love J.D., Li Y., Lazar M.A., Neuhaus D., Schwabe J.W. (2005). Structural insights into the interaction and activation of histone deacetylase 3 by nuclear receptor corepressors. Proc. Natl. Acad. Sci. USA.

[27] Delcuve G.P., Khan D.H., Davie J.R. (2012). Roles of histone deacetylases in epigenetic regulation: Emerging paradigms from studies with inhibitors. Clin. Epigenet..

[28] Roos W.P., Krumm A. (2016). The multifaceted influence of histone deacetylases on DNA damage signalling and DNA repair. Nucleic Acids Res..

[29] Eckschlager T., Plch J., Stiborova M., Hrabeta J. (2017). Histone Deacetylase Inhibitors as Anticancer Drugs. Int. J. Mol. Sci..

[30] Pojani E., Barlocco D. (2020). Romidepsin (FK228), An Histone Deacetylase Inhibitor, and its Analogues in Cancer Chemotherapy. Curr. Med. Chem..

[31] Trapani D., Esposito A., Criscitiello C., Mazzarella L., Locatelli M., Minchella I., Minucci S., Curigliano G. (2017). Entinostat for the treatment of breast cancer. Expert Opin. Investig. Drugs.

[32] Malvaez M., McQuown S.C., Rogge G.A., Astarabadi M., Jacques V., Carreiro S., Rusche J.R., Wood M.A. (2013). HDAC3-selective inhibitor enhances extinction of cocaine-seeking behavior in a persistent manner. Proc. Natl. Acad. Sci. USA.

[33] Hoffmann M.J., Koutsogiannouli E., Skowron M.A., Pinkerneil M., Niegisch G., Brandt A., Stepanow S., Rieder H., Schulz W.A. (2016). The New Immortalized Uroepithelial Cell Line HBLAK Contains Defined Genetic Aberrations Typical of Early Stage Urothelial Tumors. Bladder Cancer.

[34] Sassenberg M., Droop J., Schulz W.A., Dietrich D., Loick S.M., Wiek C., Scheckenbach K., Gaisa N.T., Hoffmann M.J. (2019). Upregulation of the long non-coding RNA CASC9 as a biomarker for squamous cell carcinoma. BMC Cancer.

[35] Jaguva Vasudevan A.A., Hoffmann M.J., Beck M.L.C., Poschmann G., Petzsch P., Wiek C., Stuhler K., Kohrer K., Schulz W.A., Niegisch G. (2019). HDAC5 Expression in Urothelial Carcinoma Cell Lines Inhibits Long-Term Proliferation but Can Promote Epithelial-to-Mesenchymal Transition. Int. J. Mol. Sci..

[36] Shechter D., Dormann H.L., Allis C.D., Hake S.B. (2007). Extraction, purification and analysis of histones. Nat. Protoc..

[37] Robertson A.G., Kim J., Al-Ahmadie H., Bellmunt J., Guo G., Cherniack A.D., Hinoue T., Laird P.W., Hoadley K.A., Akbani R. (2017). Comprehensive Molecular Characterization of Muscle-Invasive Bladder Cancer. Cell.

[38] Cerami E., Gao J., Dogrusoz U., Gross B.E., Sumer S.O., Aksoy B.A., Jacobsen A., Byrne C.J., Heuer M.L., Larsson E. (2012). The cBio cancer genomics portal: An open platform for exploring multidimensional cancer genomics data. Cancer Discov..

[39] Gao J., Aksoy B.A., Dogrusoz U., Dresdner G., Gross B., Sumer S.O., Sun Y., Jacobsen A., Sinha R., Larsson E. (2013). Integrative analysis of complex cancer genomics and clinical profiles using the cBioPortal. Sci. Signal.

[40] Chou T.C. (2010). Drug combination studies and their synergy quantification using the Chou-Talalay method. Cancer Res..

[41] Knowles M.A., Hurst C.D. (2015). Molecular biology of bladder cancer: New insights into pathogenesis and clinical diversity. Nat. Rev. Cancer.

[42] Hans F., Dimitrov S. (2001). Histone H3 phosphorylation and cell division. Oncogene.

[43] Friedrich A., Assmann A.S., Schumacher L., Stuijvenberg J.V., Kassack M.U., Schulz W.A., Roos W.P., Hansen F.K., Pflieger M., Kurz T. (2020). In Vitro Assessment of the Genotoxic Hazard of Novel Hydroxamic Acid- and Benzamide-Type Histone Deacetylase Inhibitors (HDACi). Int. J. Mol. Sci..

[44] Podhorecka M., Skladanowski A., Bozko P. (2010). H2AX Phosphorylation: Its Role in DNA Damage Response and Cancer Therapy. J. Nucleic Acids.

[45] Isono M., Hoffmann M.J., Pinkerneil M., Sato A., Michaelis M., Cinatl J., Niegisch G., Schulz W.A. (2017). Checkpoint kinase inhibitor AZD7762 strongly sensitises urothelial carcinoma cells to gemcitabine. J. Exp. Clin. Cancer Res..

[46] Patil M., Pabla N., Dong Z. (2013). Checkpoint kinase 1 in DNA damage response and cell cycle regulation. Cell Mol. Life Sci..

[47] Hirai H., Arai T., Okada M., Nishibata T., Kobayashi M., Sakai N., Imagaki K., Ohtani J., Sakai T., Yoshizumi T. (2010). MK-1775, a small molecule Wee1 inhibitor, enhances anti-tumor efficacy of various DNA-damaging agents, including 5-fluorouracil. Cancer Biol. Ther..

[48] Eot-Houllier G., Fulcrand G., Watanabe Y., Magnaghi-Jaulin L., Jaulin C. (2008). Histone deacetylase 3 is required for centromeric H3K4 deacetylation and sister chromatid cohesion. Genes Dev..

[49] Stengel K.R., Hiebert S.W. (2015). Class I HDACs Affect DNA Replication, Repair, and Chromatin Structure: Implications for Cancer Therapy. Antioxid. Redox Signal..

[50] Sanchez-Vega F., Mina M., Armenia J., Chatila W.K., Luna A., La K.C., Dimitriadoy S., Liu D.L., Kantheti H.S., Saghafinia S. (2018). Oncogenic Signaling Pathways in The Cancer Genome Atlas. Cell.

[51] Doherty S.C., McKeown S.R., McKelvey-Martin V., Downes C.S., Atala A., Yoo J.J., Simpson D.A., Kaufmann W.K. (2003). Cell cycle checkpoint function in bladder cancer. J. Natl. Cancer Inst..

[52] Castedo M., Perfettini J.L., Roumier T., Andreau K., Medema R., Kroemer G. (2004). Cell death by mitotic catastrophe: A molecular definition. Oncogene.

[53] Zhang X.H., Rao M., Loprieato J.A., Hong J.A., Zhao M., Chen G.Z., Humphries A.E., Nguyen D.M., Trepel J.B., Yu X. (2008). Aurora A, Aurora B and survivin are novel targets of transcriptional regulation by histone deacetylase inhibitors in non-small cell lung cancer. Cancer Biol. Ther..

[54] Brazelle W., Kreahling J.M., Gemmer J., Ma Y., Cress W.D., Haura E., Altiok S. (2010). Histone deacetylase inhibitors downregulate checkpoint kinase 1 expression to induce cell death in non-small cell lung cancer cells. PLoS ONE.

[55] Cornago M., Garcia-Alberich C., Blasco-Angulo N., Vall-Llaura N., Nager M., Herreros J., Comella J.X., Sanchis D., Llovera M. (2014). Histone deacetylase inhibitors promote glioma cell death by G2 checkpoint abrogation leading to mitotic catastrophe. Cell Death Dis..

[56] Goder A., Emmerich C., Nikolova T., Kiweler N., Schreiber M., Kuhl T., Imhof D., Christmann M., Heinzel T., Schneider G. (2018). HDAC1 and HDAC2 integrate checkpoint kinase phosphorylation and cell fate through the phosphatase-2A subunit PR130. Nat. Commun..

[57] Matheson C.J., Backos D.S., Reigan P. (2016). Targeting WEE1 Kinase in Cancer. Trends Pharmacol. Sci..

[58] Hanmod S.S., Wang G., Edwards H., Buck S.A., Ge Y., Taub J.W., Wang Z. (2015). Targeting histone deacetylases (HDACs) and Wee1 for treating high-risk neuroblastoma. Pediatr. Blood Cancer.

[59] Zhou L., Zhang Y., Chen S., Kmieciak M., Leng Y., Lin H., Rizzo K.A., Dumur C.I., Ferreira-Gonzalez A., Dai Y. (2015). A regimen combining the Wee1 inhibitor AZD1775 with HDAC inhibitors targets human acute myeloid leukemia cells harboring various genetic mutations. Leukemia.

[60] Bartek J., Lukas J. (2003). Chk1 and Chk2 kinases in checkpoint control and cancer. Cancer Cell.

[61] Nigg E.A. (2001). Mitotic kinases as regulators of cell division and its checkpoints. Nat. Rev. Mol. Cell Biol..

[62] Nguyen H.G., Ravid K. (2006). Tetraploidy/aneuploidy and stem cells in cancer promotion: The role of chromosome passenger proteins. J. Cell Physiol..

[63] Benada J., Macurek L. (2015). Targeting the Checkpoint to Kill Cancer Cells. Biomolecules.

[64] Saini P., Li Y., Dobbelstein M. (2015). Wee1 is required to sustain ATR/Chk1 signaling upon replicative stress. Oncotarget.

