# Supplementary figures and images for: Downregulation of Cell Cycle and Checkpoint Genes by Class I HDAC Inhibitors Limits Synergism with G2/M Checkpoint Inhibitor MK-1775 in Bladder Cancer Cells
Source: Genes (Basel). 2021 Feb 11;12(2):260. doi: 10.3390/genes12020260 (PMC7916885; doi:10.3390/genes12020260)

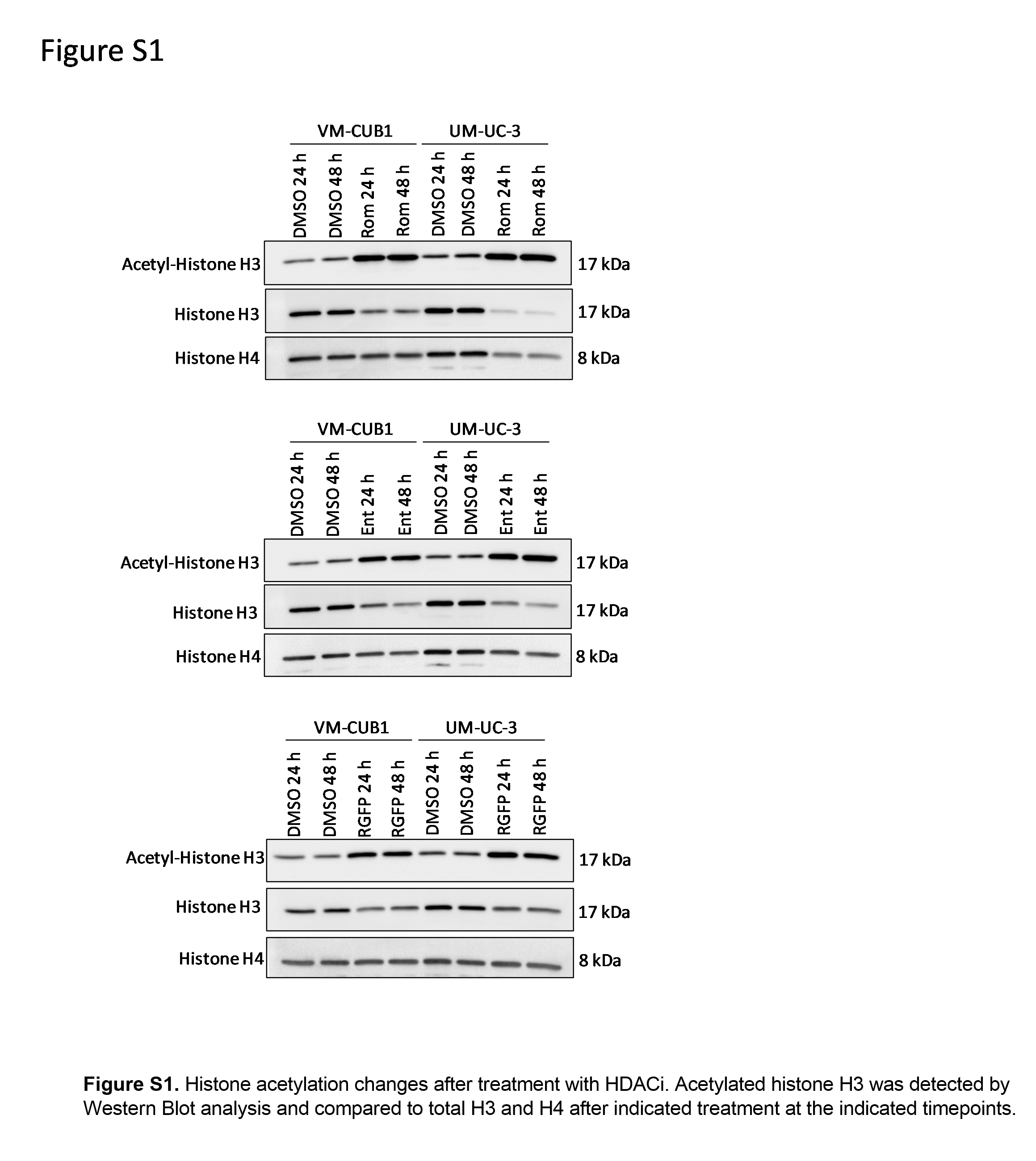

Supplement: Supplementary file 1 [file genes-12-00260-s001.zip › Supplementary/supp Figures/Figure S1.tif]

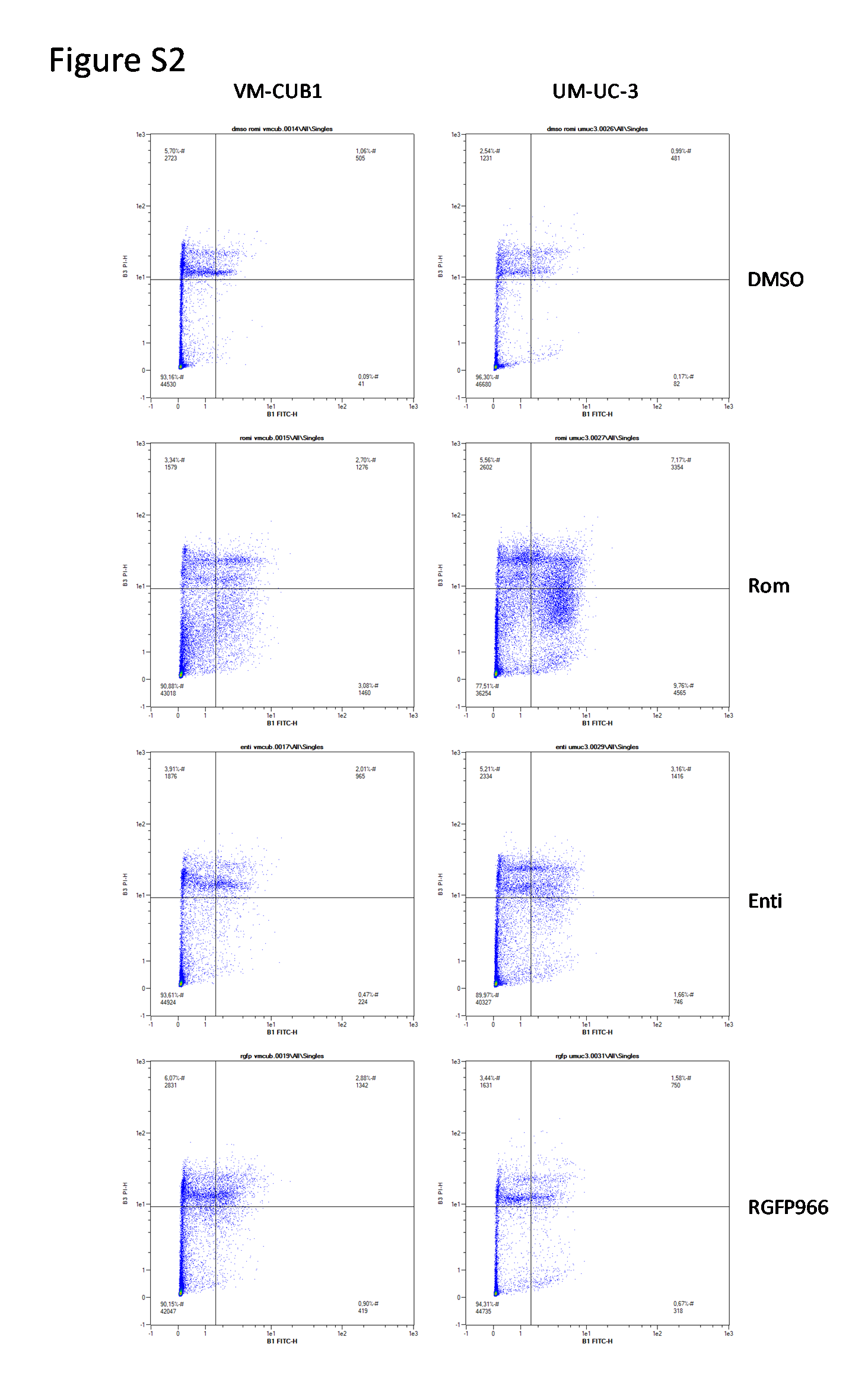

Supplement: Supplementary file 1 [file genes-12-00260-s001.zip › Supplementary/supp Figures/Figure S2.tif]

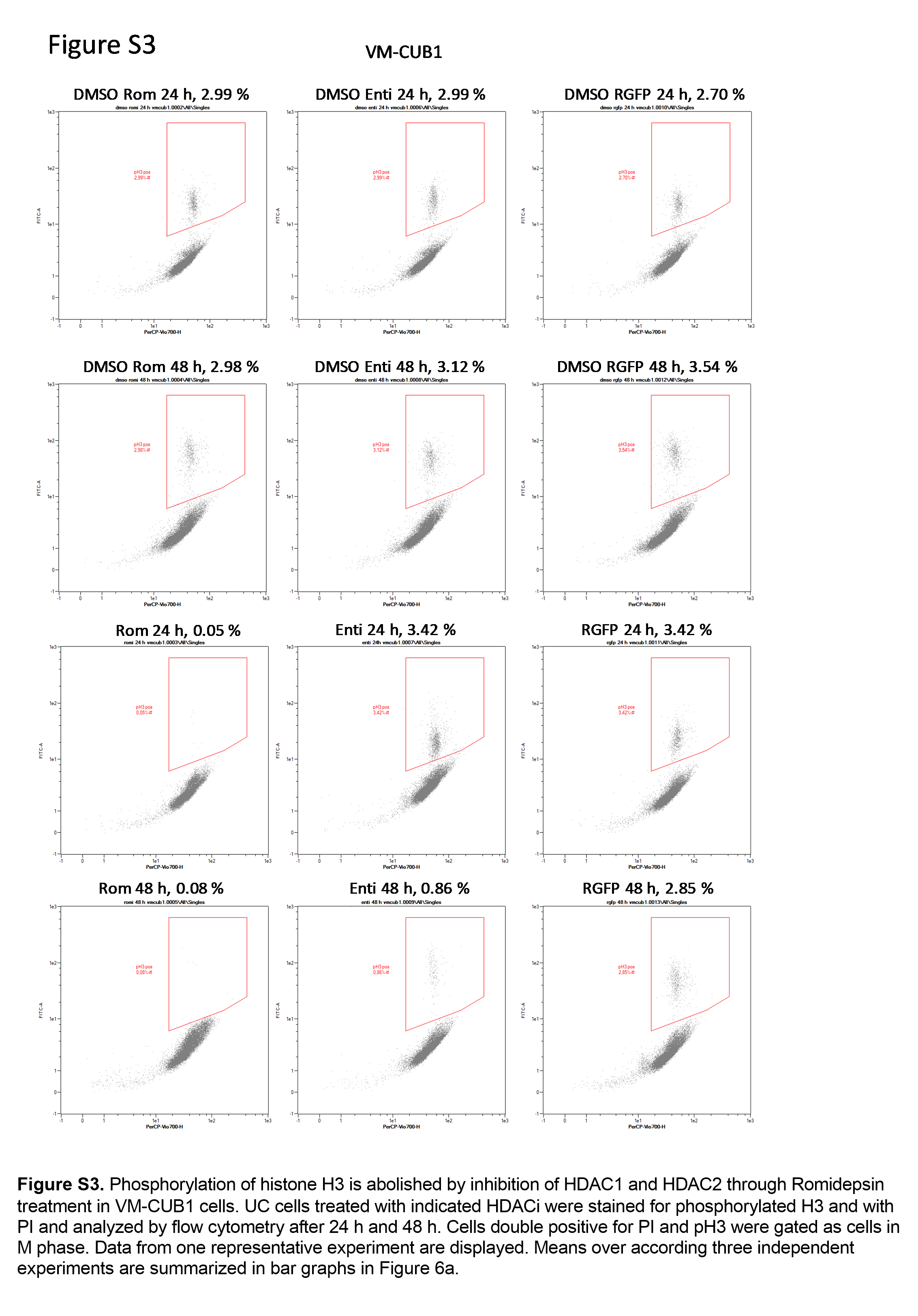

Supplement: Supplementary file 1 [file genes-12-00260-s001.zip › Supplementary/supp Figures/Figure S3.tif]

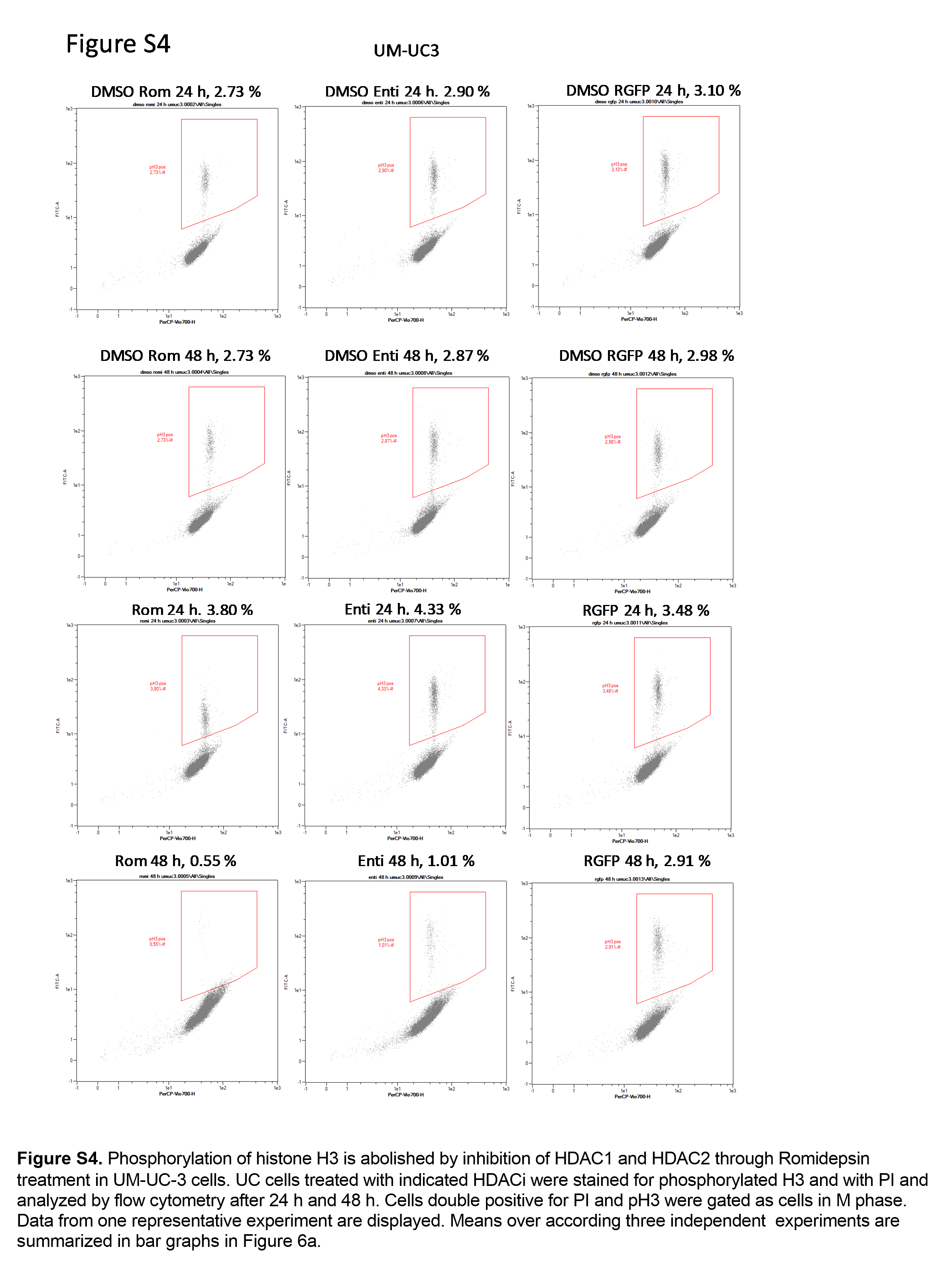

Supplement: Supplementary file 1 [file genes-12-00260-s001.zip › Supplementary/supp Figures/Figure S4.tif]

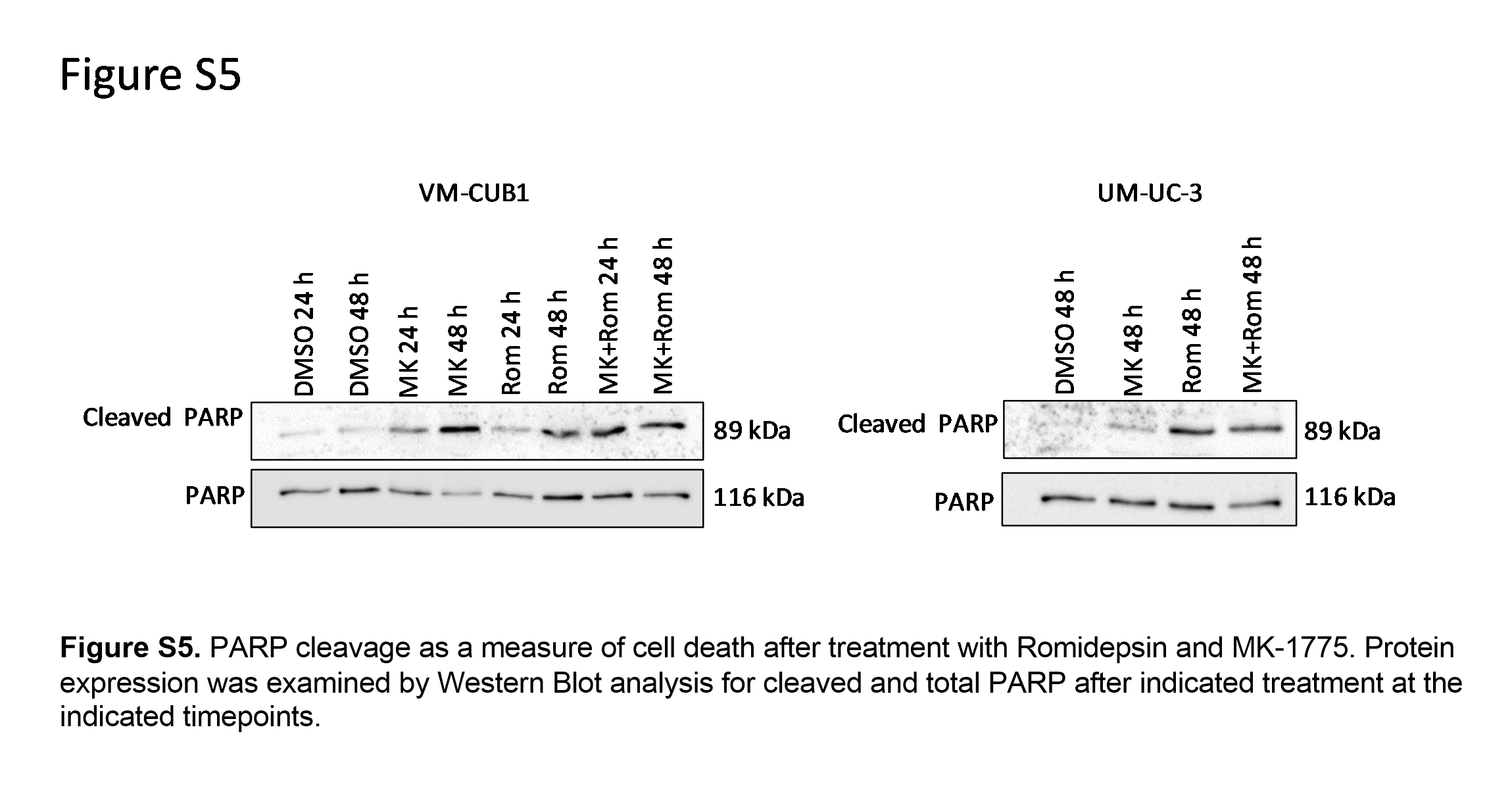

Supplement: Supplementary file 1 [file genes-12-00260-s001.zip › Supplementary/supp Figures/Figure S5.tif]

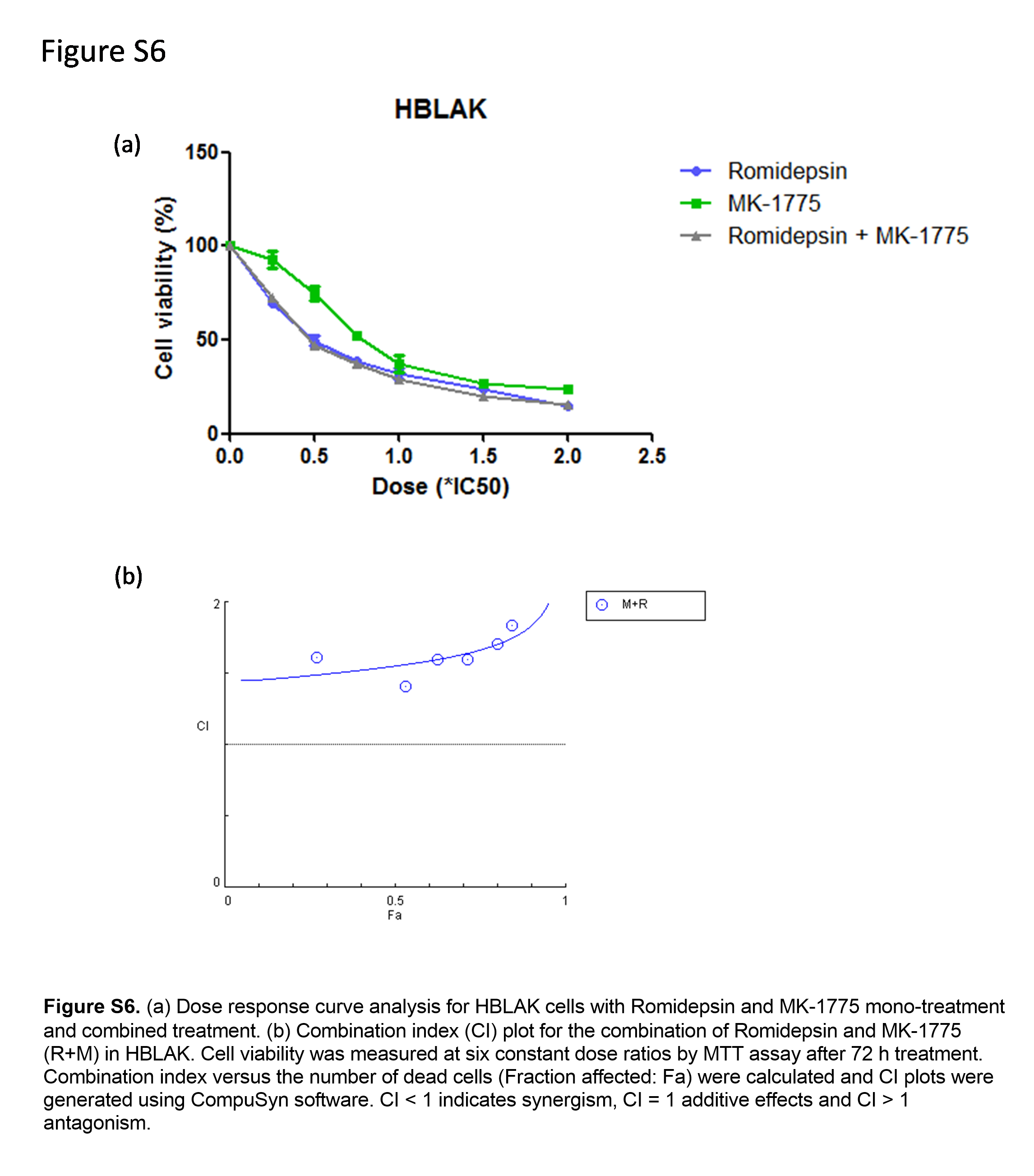

Supplement: Supplementary file 1 [file genes-12-00260-s001.zip › Supplementary/supp Figures/Figure S6.tif]
